# A Classification System in the Massive Weight Loss Patient Based on Skin Lesions and Activity of Daily Living

**Published:** 2012-02-06

**Authors:** Raffi Gurunluoglu, Susan A. Williams, Jeffrey L. Johnson

**Affiliations:** Plastic and Reconstructive Surgery, Denver Health Medical Center, University of Colorado Health Sciences, Denver.

## Abstract

**Objective:** Massive weight loss patients frequently have recalcitrant skin rashes and/or infections in the abdominal region and interference with activities of daily living (ADL) due to the redundant abdominal tissues. **Materials and Methods:** A classification of these functional problems is described on the basis of the authors' experience with 75 consecutive massive weight loss patients undergoing surgery between March 2006 and August 2010. Patients are classified in 3 types. Type I: Chronic skin problems confined to the lower abdomen under the pannus and/or the skin folds of redundant pannus (± posterior lower torso). Type II: Chronic skin problems around the navel and/or under a secondary pannus in the mid/upper abdomen in addition to those observed in type I. Type III: Abdominal pannus and/or secondary roll in the abdomen with no chronic skin problems. These 3 types are further categorized on the basis of the abdominal pannus size and associated ADL interference by the subtypes: (A) those with a small pannus and no ADL interference (B) for the large excessive pannus and ADL interference. **Results:** Fifty-two patients (70%) were classified as type I. Fifteen patients (20%) were type II. Only 8 patients (10%) had no skin problems. Overall complication rate was 21%. Mean follow-up was 13.3 months postoperatively. **Conclusions:** The classification system is proposed to provide a practical method by which to categorize massive weight loss patients based upon the presence and location of skin problems and ADL interference. Surgical guidelines to address these functional requirements are presented on the basis of the classification system.

In the last decade, there has been a dramatic increase in the number of massive weight loss patients seeking body-contouring procedures as a result of the increasing incidence of bariatric surgery. The most complete statistics from the American Society for Metabolic and Bariatric Surgery estimate that 220000 bariatric procedures were performed in 2009.^1^ Total body-contouring procedures after massive weight loss reached 58669 in the 2008, according to the data obtained from the American Society of Plastic Surgeons (ASPS).^2^ Abdominal body-contouring surgeries were the second most frequently performed procedures (17478) in massive weight loss patients in 2008.^2^ In 2 separate postbariatric surgery surveys conducted by the ASPS, abdominoplasty and panniculectomy were ranked as the number 1 or 2 most commonly performed procedures.

Several authors have described techniques and classification systems for optimizing the aesthetic results of the abdominal contour surgery in massive weight loss patients.^3^^-^8 Song et al^7^ developed a scale to classify deformities in 10 different sites, including the abdomen. Wallach^8^ proposed a classification system of deformities of the abdominotorso region from types I to V.

Aesthetic dissatisfaction in the abdominotorso region is a common presentation in massive weight loss patients. However, these patients also frequently suffer from skin lesions such as intertrigo, recalcitrant rashes, sores, and dermatitis in the abdominal region and interference with activities of daily living (ADL) due to their excessive abdominal tissues.

To our knowledge, no classification system exists on the basis of these functional problems in treating the abdominal region of the massive weight loss patient. The authors propose a new classification system with surgical guidelines founded on these functional requirements. Included are patient examples and a discussion of the usefulness of this classification system.

## MATERIALS AND METHODS

We analyzed our experience in treating 75 consecutive massive weight loss patients who underwent surgery between March 2006 and August 2010 at Denver Health Medical Center, University of Colorado Health Sciences Center. Patients' ages ranged from 27 to 62 years. Seventy-three of our patients were female. Our patients who adopted methods of weight loss included 60 patients who underwent open gastric bypass, 8 patients who implemented lifestyle and dietary changes, and 7 patients who underwent lap band procedure. The average body mass index score of the patients before weight loss measures was 54.26 with a range of 88.39 to 36.15. The average body mass index score after weight loss and immediately before panniculectomy was 30.61 with a range of 47 to 18.97. Overall, the average weight loss for our patients was 138 lb with weight loss ranging from 319 to 60 lb in our sample. Sixteen patients had incisional ventral hernia.

### Classification system and functional guidelines

Preoperatively, patients are evaluated for the extent and location of persistent skin lesions in the abdomen, their relation with skin/fat redundancy and position of the umbilicus, size of the excessive abdominal tissue and associated interference with ADL, and for the presence of hernias.

Information on ADL is retrieved from the 36-Item Short Form Health Survey (SF-36), which is routinely given to our massive weight loss patients both at the time of preoperative evaluation and in the postoperative phase.^9^ This survey includes questions about physical functioning and evaluation of one's ability to perform physical activity. Specifically, in the physical functioning scoring section of the SF-36, ADL such as the ability to perform personal hygiene, dress/undress, and general mobility are addressed along with the instrumental activities of daily living (IADL) such as the ability to grocery shop. In addition to factoring the physical functioning score of the SF-36, the self-report by patients and findings during observation of patients in their initial clinic visit regarding the impact of their pannus on their ability to perform ADL are considered by the surgeon. A large redundancy of skin and subcutaneous fat in the abdominal area can hinder a person's ability to maneuver during the simple actions necessary for independent living and thus decrease the quality of life. Patients report that the size of their pannus makes it difficult for them to bend or kneel, to find clothing that fits properly, and that it can be complicated for them to wash effectively underneath the skin folds. “Activities of daily living” interference is defined by the authors as at least 1 response of a 1 rating on section 3 of the SF-36 excluding question 3(a) regarding vigorous activities (Table 1).

The principal factors leading to surgery include the presence of the aforementioned skin lesions in varying locations and ADL interference associated with size of the pannus. When these factors were taken into consideration, it led us to the classification system proposed in Table 2. Patients are classified as type I or II, which is dependent on the location of abdominal skin lesions such as intertrigo, rashes, infections, and ulceration that are unresponsive to conservative therapy. Type I patients have their skin problems confined to only the lower abdomen under the pannus and/or in the skin folds of the redundant pannus. On the contrary, type II patients have additional skin problems around the navel and/or under a secondary pannus in the mid/upper abdomen. In both types I and II, patients may also additionally experience intertrigo in the posterior lower torso region. A third type (type III) is classified on the basis of presence of a pannus but without concomitant skin problems. The interference with ADL associated with overall size of the excessive skin and subcutaneous fat in the abdomen is integrated in each of these 3 types as A (small and no ADL interference) and B (large and ADL interference).

Based on the classification system, functional surgical guidelines are described (Table 3). In the authors' experience based on the findings of the present case series, posterior deformities were infrequently associated with chronic skin problems. However, some massive weight loss patients may have intertrigo in the posterior torso especially along the superior buttock cleft. Therefore, excisions can be extended to include the redundant skin and subcutaneous fat in the posterior lower torso to address symptomatic skin, if necessary.

## REPRESENTATIVE CASES

### Patient 1

**Figure 1:**
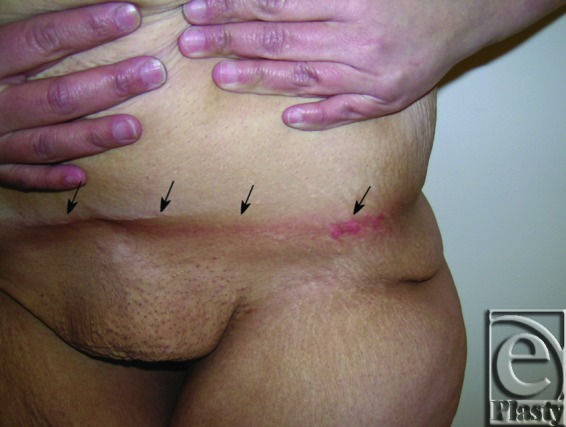
Frontal view of patient 1 (type IA) demonstrating skin sores in her bilateral groins (arrows) and suprapubic region (arrows) secondary to excess overhanging skin. An infraumbilical panniculectomy with umbilical transposition was adequate to treat her skin problems.

**Figure 2:**
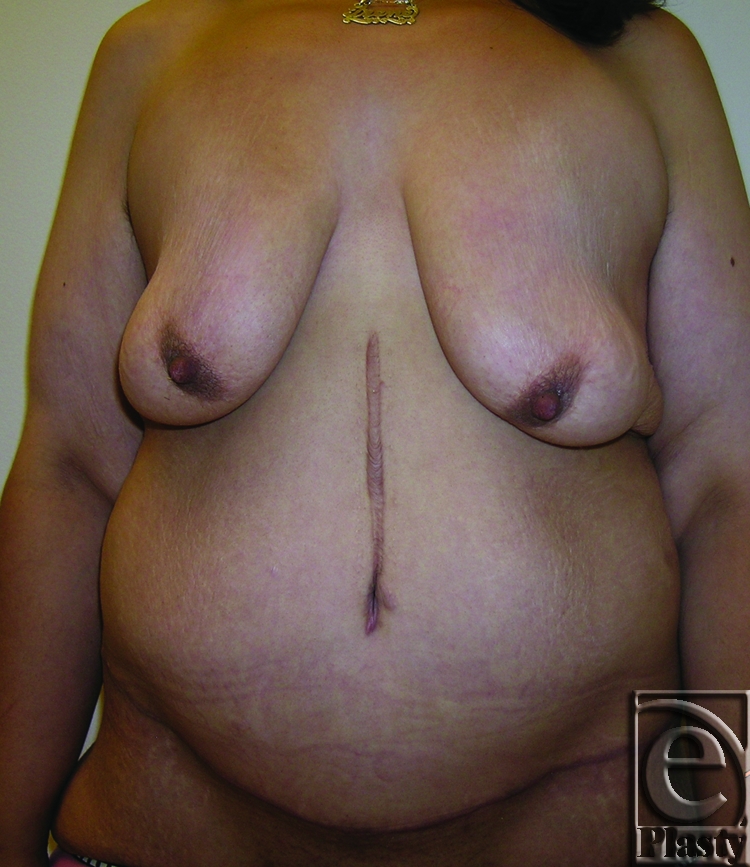
Patient 1 at 13-month follow-up showed complete resolution of the skin problems that she had prior to surgery.

This was a 32-year-old female patient who lost 219 lb after gastric bypass surgery. Her postbariatric weight was 185 lb. She had persistent skin sores bilaterally in her groin and also in her suprapubic region secondary to excess overhanging skin. No ADL interference was diagnosed. She was classified as type IA (Fig 1). An infraumbilical panniculectomy with the need for umbilical transposition was carried out. Her 13-month follow-up showed complete resolution of the skin problems that she had prior to surgery (Fig 2).

### Patient 2

**Figure 3:**
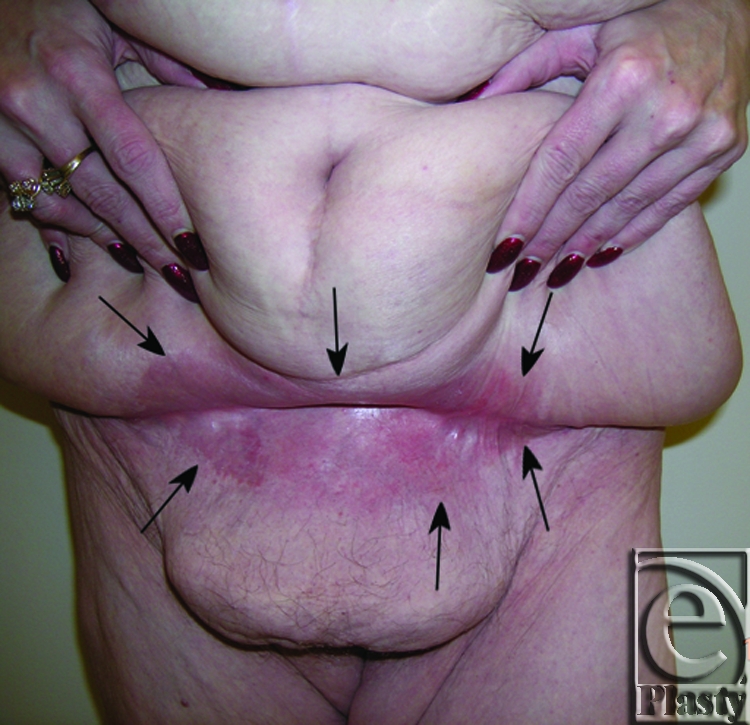
Frontal view of patient 2 (type IB) with a large pannus lifted up by the patient to demonstrate persistent skin rash (arrows) in the lower abdomen and suprapubic area. She underwent a horizontal panniculectomy with umbilical sacrifice.

**Figure 4:**
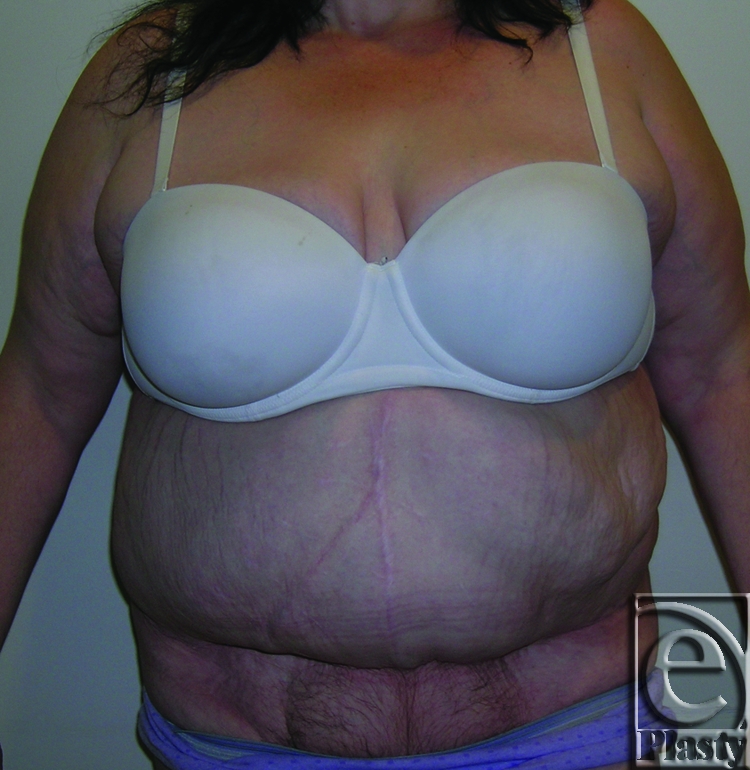
Postoperative frontal view of patient 2: Follow-up at 12 months showed resolution of her skin problems in the lower abdomen and improved activities of daily living.

This was a 44-year-old female patient who lost 180 lb after gastric bypass surgery. At presentation she weighed 200 lb and had chronic skin problems under her pannus in the suprapubic region. In addition, she had ADL interference due to the size of her pannus and was classified as type IB (Fig 3). She underwent a horizontal panniculectomy with umbilical sacrifice. Follow-up at 12 months showed resolution of her skin problems in the lower abdomen and improved ADL (Fig 4).

### Patient 3

**Figure 5:**
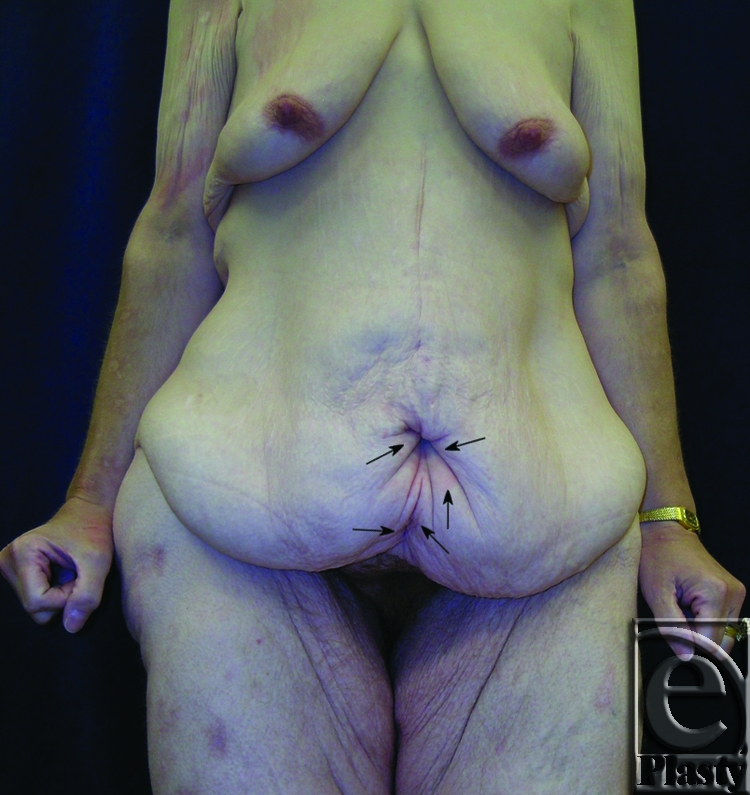
Frontal view of patient 3 (type IIA) who has a small pannus with skin rashes around the navel (arrows) and in the lower abdomen (arrows). A horizontal panniculectomy with navel transposition was performed.

**Figure 6:**
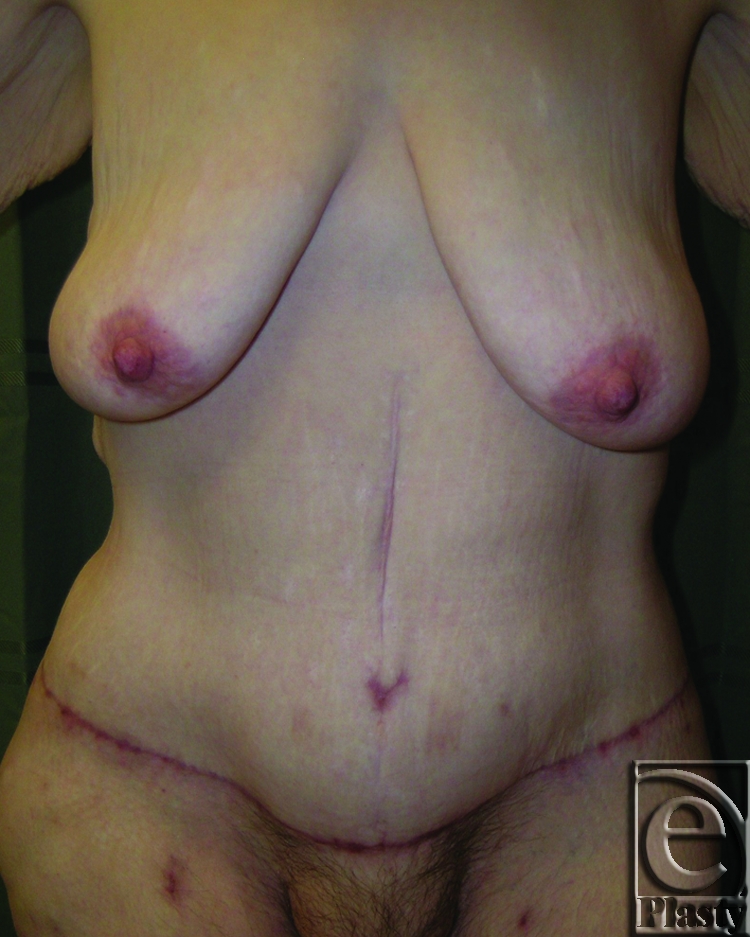
Postoperative frontal view of patient 3 demonstrated no skin problems at 9-month follow-up.

This was a 48-year-old female patient who lost 160 lb after gastric bypass surgery. Her weight at presentation was 170 lb. She had a recalcitrant intertrigo in the skin around her navel and in her lower abdomen but did not have ADL interference. She was classified as type IIA (Fig 5). The patient wanted to preserve her navel. A horizontal panniculectomy and navel transposition following minimal abdominal undermining were carried out. Her follow-up at 9 months demonstrated no skin problems (Fig 6).

### Patient 4

**Figure 7:**
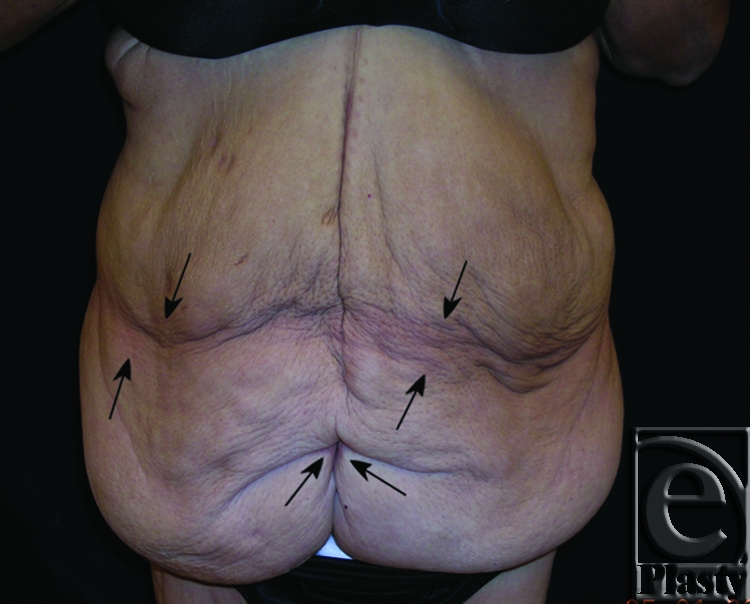
Frontal view of patient 4 (type IIB) with a large pannus. Arrows showing persistent skin problems around her navel and also in the mid-abdomen. A high-level horizontal panniculectomy with a minimal vertical skin excision and umbilical sacrifice was performed.

**Figure 8:**
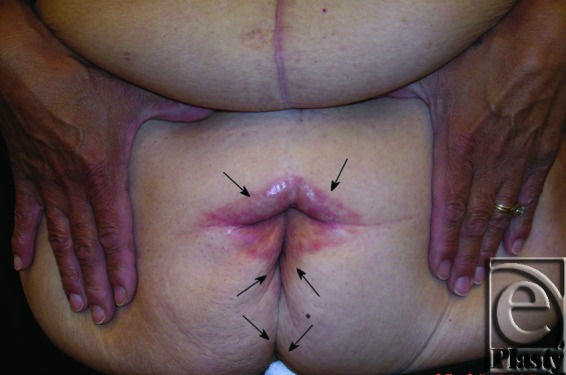
Close-up view demonstrating the extensive skin rash around her navel (arrows) and in the lower abdomen (arrows) in patient 4.

**Figure 9:**
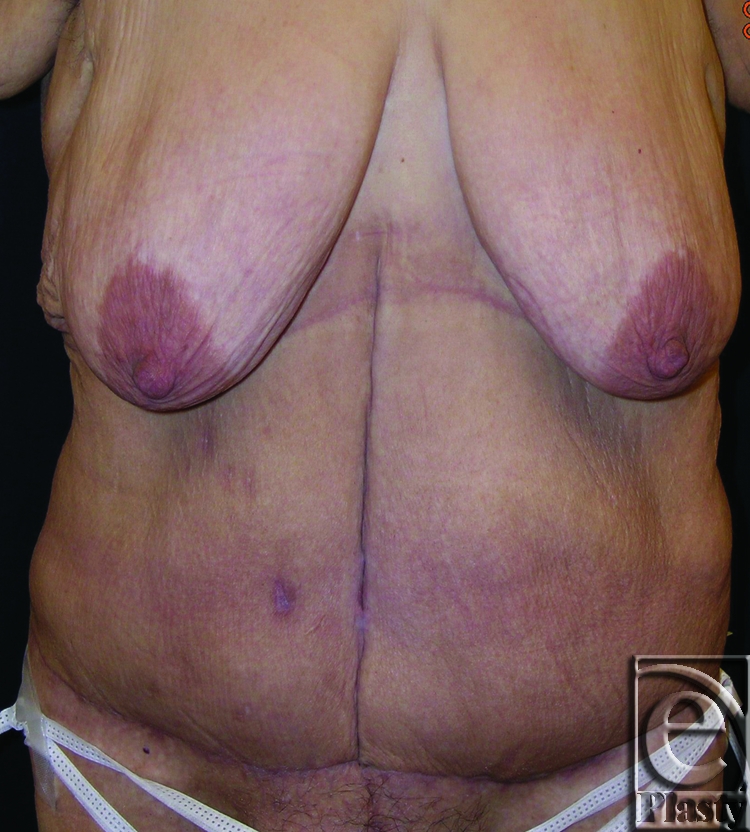
Postoperative frontal view of patient 4. Her follow-up at 14 months demonstrated no skin problems and improved ADL.

This was a 40-year-old female patient who lost 130 lb after gastric bypass surgery. She weighed 195 lb when she presented with a persistent rash in the skin around her navel and lower abdomen. She also had a significant abdominal redundancy and associated interference with ADL. She was classified as type IIB (Figs 7 and 8). A horizontal panniculectomy, combined with a minimal vertical redundant skin excision and umbilical sacrifice, was performed. Her follow-up at 14 months demonstrated no skin problems and improved ADL (Fig 9).

### Patient 5

**Figure 10:**
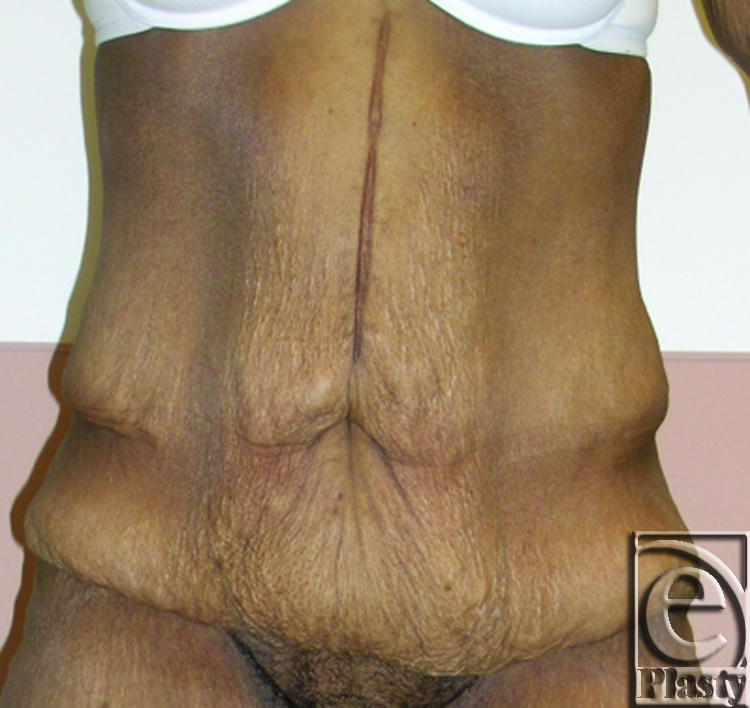
Frontal view of patient 5 (type IIIA) with a small pannus. She underwent a fleur-de-lis abdominoplasty with umbilical transposition and fascial plication.

**Figure 11:**
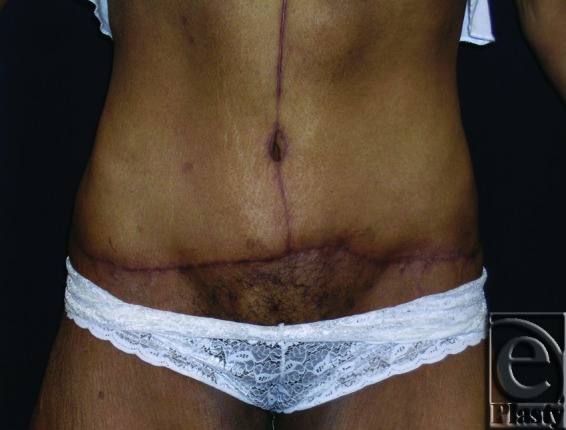
Postoperative frontal view of patient 5 at 11 months. The patient was pleased with the outcome.

This 43-year-old female patient had a history of depression and osteoarthritis of both knees. She underwent gastric bypass surgery and lost 230 lb. She presented with redundant abdominal skin. She did not have any skin problems or interference with ADL. She was classified as type IIIA (Fig 10). A cosmetic abdominoplasty with fleur-de-lis pattern, fascial plication, and umbilical transposition was performed. The patient was pleased with the outcome at 11-month follow-up after surgery (Fig 11).

### Patient 6

**Figure 12:**
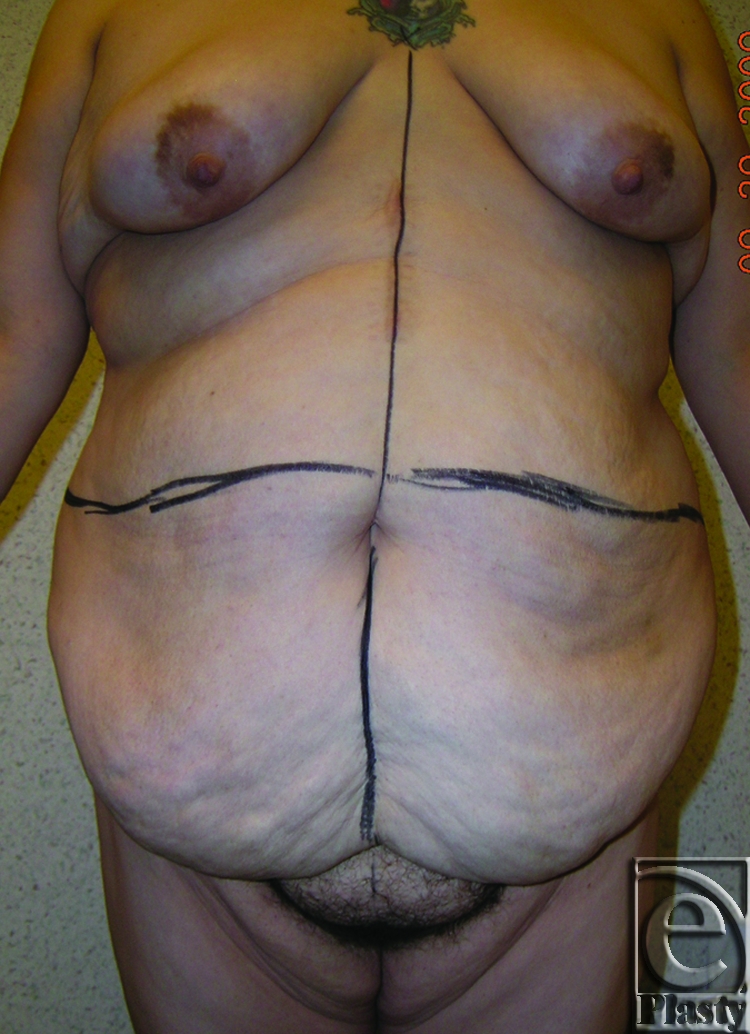
Frontal view of patient 6 (type IIIB). She had a large pannus with no skin problems but interference with activities of daily living. An abdominoplasty with umbilical transposition was performed.

**Figure 13:**
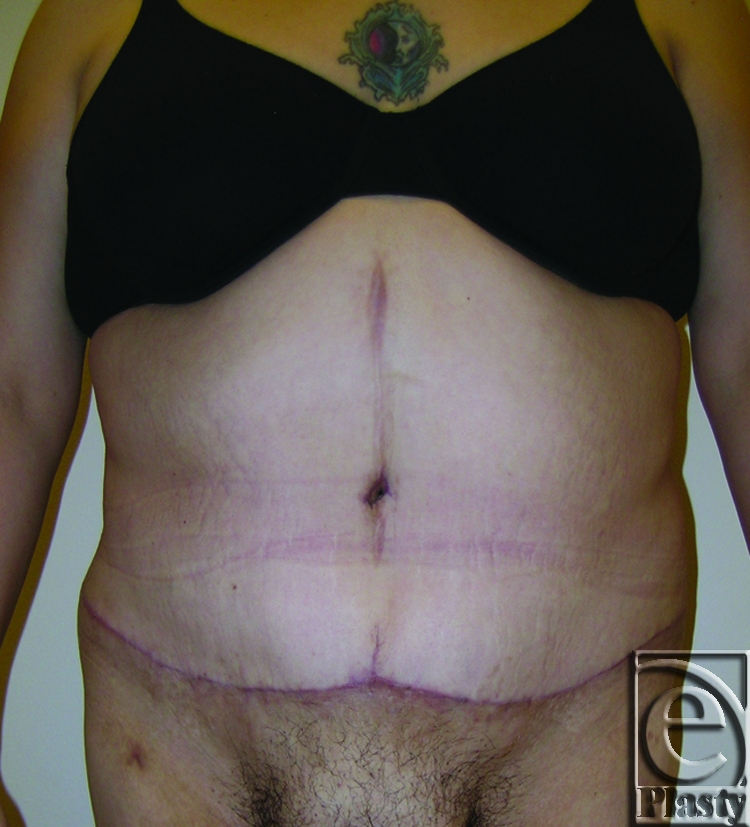
Postoperative frontal view of patient 6 who was satisfied with improved activities of daily living at 10 months after surgery.

This 37-year-old female patient with a history of hypertension presented with a significant redundant abdominal pannus after weight loss of 90 lb following gastric bypass surgery. Her weight was 190 lb at presentation. She did not have any skin problems but described difficulty with ADL. She was classified as type IIIB (Fig 12). She wanted to preserve her navel. She underwent a horizontal panniculectomy with navel transposition and her diastasis recti was repaired by fascial plication. The patient was satisfied with improved ADL at 10 months after surgery (Fig 13).

## RESULTS

Seventy-five massive weight loss patients underwent surgery. Fifty-two patients (70%) were classified as type I. Of these patients, 26 had interference with ADL due to large pannus (type IB). Twenty percent (15) of patients were type II. Eight patients were classified as type IIB. Only 8 patients (10%) had no skin problems. Three of these patients were classified as type IIB due to interference in ADL. Sixty-five patients (86%) received umbilical transposition. Sixteen patients had a concomitant incisional ventral hernia repair. Mean follow-up was 13.3 months after the surgery. Skin signs were resolved in all but 1 patient having persistent problems prior to surgery. We demonstrated improvement in postoperative ADL ranging from 15.2% to 25.7% with a mean of 20.4%. Overall complication rate was (21.0%); there were 3 cases of hematoma development of which 2 required surgical evacuation, 4 cases of seroma formation of which 2 required drainage, 4 cases of mild wound dehiscence that resolved without surgical intervention, 3 cases of cellulitis/soft tissue infection detected during follow-up requiring antibiotic treatment, one case of keloid scar formation, and one case of hernia recurrence.

## DISCUSSION

Most patients want the very best possible aesthetic and functional results, as well as preservation of their umbilicus if possible. Nevertheless, a classification system has been proposed to distinguish what is medically necessary in treating the abdominal region of massive weight loss patients. The authors recognized 3 different types of patients based on the presence or absence of intertrigo and its location in the abdomen, and interference with ADL associated with general size of the abdominal pannus. In our experience, the size of the pannus was associated with interference in ADL. The vast majority of patients reporting difficulty in ADL were those who had relatively larger abdominal tissue. In other words, none of the patients who had problems with ADL had a small-sized pannus and likewise none of the patients with a small-sized pannus reported ADL problems. Therefore, a classification into subtypes as A (small, no ADL interference) and B (large, ADL interference) was made.

While skin lesions are easy to document and diagnose, the evaluation of ADL interference is difficult to objectively quantify because of reliance upon self-report by the patient. Therefore, it is crucial for the surgeon to dedicate time and awareness of how the pannus impacts a patient's life during the preoperative visit. One must pay careful attention to not only the physical dimensions of the pannus but also how it has an effect on one's ability to ambulate. Also, it can be argued that if patients felt there was a benefit to answering ADL questions a particular way that this could cause bias in the self-reported answers on the SF-36. We also understand that lacking a severity-rating system of ADL inference is a shortcoming of our method of classification. For instance, a type III patient may be more dysfunctional than a type I or II. However, we felt that adding a severity rating would only lead to a potential complication and point of dissention.

Persistent skin problems that are unresponsive to conservative treatment are most commonly encountered in the groin skin, suprapubic skin, and/or under or on the redundant pannus in the lower abdomen. Type I, in our experience, is the most common presentation among the massive weight loss patients with skin problems. *An infraumbilical horizontal functional panniculectomy* without the need for umbilical transposition can be carried out in most type IA patients. However, if treatment of skin lesions necessitates excision of more abdominal skin and subcutaneous tissue that would result in unnatural displacement of the navel, then umbilical transposition should be considered. Type IB patients would benefit from a larger amount of tissue removal from the abdomen to help improve ADL. Therefore, umbilical transposition is usually required in these patients. However, the decision to do so would depend on adequate perfusion of the umbilicus. On the contrary, in our experience, patients with massive pannus tend not to care about preservation of their navel and thus a sacrifice decision prior to operation can sometimes be made.

Type II presentation is the next most common scenario in patients with skin problems. Additional persistent skin problems around the navel and/or under the secondary rolls in the mid-abdomen necessitate a *higher-level horizontal functional panniculectomy* that includes these problematic skin areas. A navel transposition is considered after ensuring adequate perfusion to the umbilicus by intraoperative assessment. Because of the redundant nature of the abdominal skin, minimal abdominal undermining is adequate for umbilical transposition in most cases. On the other hand, the large/massive size of the pannus in some type IIB (similar to type IB) patients may negate the ability to preserve the umbilicus, resulting in the need to sacrifice. In type II patients, rarely, there is a need for vertical redundant skin and fat excision to address the intertrigo in the vertical skin folds of the mid-abdomen or around navel and to reduce the amount of abdominal tissue interfering with ADL.

Type III is integrated into the classification system to categorize the group of massive weight loss patients who have no persistent skin problems. A variety of body-contouring procedures in the abdominotorso region can be employed in these patients. These procedures primarily address the aesthetic dissatisfaction and include cosmetic abdominoplasty, reverse abdominoplasty, fleur-de-lis approach, circumferential abdominoplasty, fascial plication, liposuction, and monsplasty. Patients with a large pannus usually suffer from interference with ADL (type IIIB) and therefore a functional panniculectomy is indicated to reduce the burden on the body in these patients.

In our experience, the majority of massive weight loss patients requested salvation of the umbilicus. We were able to preserve the umbilicus in 86% of our patients. Because of the redundant and stretchable properties of the abdominal skin and elongated nature of the umbilical stalk, only minimal abdominal undermining was required in most massive weight loss patients to allow for safe umbilical transposition.

Therefore, in regard to translocation of the umbilicus, we feel that young plastic surgeons should not be encouraged that sacrificing the umbilicus is the first option they should consider. Every attempt should be made to preserve it, especially in those patients who strongly desire their umbilicus and in patients in whom umbilical transposition can be performed without compromising its perfusion. On the contrary, in patients with significant medical comorbidities and extremely large pannus including massive cases, or in patients in whom the viability of umbilicus is compromised, umbilical sacrifice may be justified. In the present case series, umbilical sacrifice was warranted in only a minority of patients classified as type IB, IIB, and IIIB.

Despite the increasing popularity of a laparoscopic approach, many morbidly obese patients are still offered open gastric bypass surgery. Consequently, sometimes, massive weight loss patients require hernia repair (umbilical, incisional, or ventral). In our series, 21.3% of patients required concomitant hernia repair in addition to presenting with signs and symptoms associated with panniculus. This can be performed safely either in conjunction with the redundant skin/pannus excision procedure or alone in any patient type presented. In fact, concurrent panniculectomy has been shown to minimize the risk of hernia recurrence.^10^^-^12 Therefore, this approach would be beneficial particularly in type IB, IIB, and IIIB patients who have a large pannus. These findings have also been supported in our study. We believe that the proposed classification system would have several benefits.

As the number of massive weight loss patients who have undergone bariatric procedure under insurance coverage increases, more and more patients are seeking reconstructive surgery, and determination of medical necessity has become more important then ever especially in patients with insurance. Therefore, the use of such a classification method would be helpful in increasing the recognition of the medical and functional problems and their accurate documentation in massive weight loss patients. Furthermore, this classification would help surgeons and third-party payers differentiate between aesthetic and functional needs of the abdominal region. Currently, third-party payers in the United States utilize medical necessity guidelines only for excessive panniculus and associated skin problems in the lower abdomen.^13^^,^^14^ It is almost impossible to obtain an approval for postbariatric reconstructive surgery with no clinical evidence of skin lesions. On the contrary, the majority of postbariatric procedures are paid by the insurance, regardless of the presence of skin lesions in other countries especially in Europe.

The infraumbilical horizontal panniculectomy without umbilical transposition has been proposed as the sole method of treating the aforementioned signs and symptoms among third-party payers in the United States. There are no guidelines defined for recalcitrant skin problems except for those in the lower abdomen. In addition, interference with ADL associated with an excessive pannus has not been classified as a medical necessity criterion alone for a functional panniculectomy despite the fact that this is accepted as a medical indication by the ASPS.^15^

On the basis of this patient classification system, we demonstrate that there are more functional procedures that may be required to treat chronic skin problems that occur in areas other than lower abdomen such the mid-abdomen and around the navel. In addition, interference with ADL due to excessive abdominal tissues should be taken into surgical consideration to improve the functionality in the massive weight loss patient. This would be particularly beneficial in patients having additional hip/knee/back problems, which are not uncommon in massive weight loss patients.

Although there is some overlap in the surgical treatment of symptomatic massive weight loss patients in our classification system, we feel that these are the best surgical procedures to address the skin involvement of the abdomen associated with the abdominal redundancy. We encourage the use of the classification system for outcome analysis in massive weight loss patients by prospective clinical studies to validate its reliability.

While our classification system and suggested procedures address the concerns of intertrigo and ADL interference, it is our goal to obtain the best overall contour possible in every case. However, insurance coverage, patient's financial status, and patient-related comorbidities may become limiting factors in performing more involved procedures necessary to obtain the best aesthetic outcome in some massive weight loss patients.

## Figures and Tables

**Table 1:** Activities of daily living (ADL) questions from Section 3 of the 36-Item Short Form Health Survey*

ADL specific questions on the Short Form 36—Section 3
Moderate activities, such as moving a table, pushing a vacuum cleaner, bowling, or playing golf
Lifting or carrying groceries
Climbing several flights of stairs
Climbing one flight of stairs
Bending, kneeling, or stooping
Walking more than a mile
Walking several blocks
Walking one block
Bathing or dressing yourself

*Authors' definition of ADL interference is at least 1 response of a 1 rating in section 3 excluding question 3(a) regarding vigorous activities

**Table 2:** Classification system for patients with pannus following massive weight loss based on skin involvement, size of pannus, and activities of daily living (ADL) interference

Type	Location of skin involvement	Size of pannus	ADL interference*
I A	Lower abdomen ± posterior lower torso	Small	No ADL interference
I B	Lower abdomen ± posterior lower torso	Large	ADL interference
II A	Lower abdomen and around umbilicus, and/or under a secondary pannus in mid/upper abdomen ± posterior lower torso	Small	No ADL interference
II B	Lower abdomen and around umbilicus, and/or under a secondary pannus in mid/upper abdomen ± posterior lower torso	Large	ADL interference
III A	No skin involvement	Small	No ADL interference
III B	No skin involvement	Large	ADL interference

*ADL interference is defined by the authors as at least 1 response of a 1 rating on section 3 of the SF 36 excluding question 3(a) regarding vigorous activities.

**Table 3:** Surgical guidelines for the classification system

Classification type	Procedure	Intraoperative assessment of perfusion of umbilicus	Umbilical transposition
**Type I A**	Infraumbilical panniculectomy ± Posterior lower torso dermolipectomy	NA	No need
**Type I A/B**	Panniculectomy ± Posterior lower torso dermolipectomy	Adequate Inadequate	Yes No
**Type II A/B**	Panniculectomy ± Vertical component ± Posterior lower torso dermolipectomy	Adequate Inadequate	Yes No
**Type III A**	Aesthetic abdominoplasty	Adequate Inadequate	Yes No
**Type III B**	Panniculectomy	Adequate Inadequate	Yes No
